# Excitatory repetitive transcranial magnetic stimulation to left dorsal premotor cortex enhances motor consolidation of new skills

**DOI:** 10.1186/1471-2202-10-72

**Published:** 2009-07-07

**Authors:** Lara A Boyd, Meghan A Linsdell

**Affiliations:** 1 Department of Physical Therapy, University of British Columbia, Vancouver, Canada; 2 Brain Research Centre, University of British Columbia, Vancouver, Canada; 3 Graduate Program in Rehabilitation Sciences, University of British Columbia, Vancouver, Canada

## Abstract

**Background:**

Following practice of skilled movements, changes continue to take place in the brain that both strengthen and modify memory for motor learning. These changes represent motor memory consolidation a process whereby new memories are transformed from a fragile to a more permanent, robust and stable state. In the present study, the neural correlates of motor memory consolidation were probed using repetitive transcranial magnetic stimulation (rTMS) to the dorsal premotor cortex (PMd). Participants engaged in four days of continuous tracking practice that immediately followed either excitatory 5 HZ, inhibitory 1 HZ or control, sham rTMS. A delayed retention test assessed motor learning of repeated and random sequences of continuous movement; no rTMS was applied at retention.

**Results:**

We discovered that 5 HZ excitatory rTMS to PMd stimulated motor memory consolidation as evidenced by off-line learning, whereas only memory stabilization was noted following 1 Hz inhibitory or sham stimulation.

**Conclusion:**

Our data support the hypothesis that PMd is important for continuous motor learning, specifically via off-line consolidation of learned motor behaviors.

## Background

It is clear that skilled practice is essential for the acquisition of learned motor behaviors [1-3] and that the brain continues to process information from practice sessions well beyond the timeframe of motor performance [4-7]. In fact, many changes take place after practice that both strengthen and modify the motor skill being learned. These changes represent motor memory consolidation [5-7] a process whereby new, fragile memories are transformed into more permanent, robust and stable state.

Consolidation of motor skill memories has been purported to take two forms: 1) off-line improvements in behavior that occur in between practice sessions, and 2) memory stabilization which reduces the fragility or susceptibility to interference by other motor actions while behavioral improvements are maintained [6-9]. While these two elements of motor consolidation are not completely independent of one another, the degree to which they interact and/or rely of unique neural structures remains unclear.

Functional brain imaging has been used to consider how the neural structures associated with movement change as motor learning occurs [5,10,11]. Following practice, while motor ability remains unchanged, positron emission tomography shows that the brain recruits new regions to perform the task. Early in motor skill acquisition prefrontal brain regions are active. Later, there is a shift in activation to premotor, posterior parietal and cerebellar structures [5]. Evolution of the network activated in association with motor learning is widely believed to support motor consolidation or the increase in stability of the new skill [5-7,11].

Intracortical recordings in animals and human neuroimaging studies indicate that the premotor cortex (PMC) plays an important role in the selection of movements [12,13]. The PMC can be functionally segregated according to the type of movement being selected [14-18]. The ventral premotor (PMv) cortex is involved in grasping movements that are externally triggered by the environment [12], while the dorsal premotor (PMd) cortex appears to be particularly important in the selection movements that are learned [12,19].

At present it is unclear whether PMd is important for selecting learned movements [20] or for learning new movements [21,22]. Some animal work suggests that PMd is involved in motor learning. The PMd cortex may be critical for holding sensory information in working memory and then converting it to a motor program [22]. Single cell recordings from PMd during motor task practice demonstrate the emergence of new motor programs that are based on the sensory information acquired through practice [22]. Other research in humans, suggests a dominance of left PMd for the selection of learned movements [12,19]. Functional magnetic resonance imaging (MRI) studies show that only right PMd is active during movements of the left hand; however, left PMd is activated during movements of both the right and left hands [19]. The disparate nature of PMd activity during movements of each hand has also been confirmed by transcranial magnetic stimulation (TMS) studies demonstrating that disruption of left PMd alters movements in both hands [21].

Though past work has purported to investigate the role of PMd in skill acquisition it has often been limited by the failure to consider motor learning at a separate delayed retention test [7,23] but rather has largely considered behavioral changes across a single day or session. Without data from separate sessions it is impossible to evaluate the impact of any intervention of the long-term, permanent ability to perform new motor skills [24]. Because we were interested in the possibility that PMd might play a role in consolidation of new motor learning we designed the present experiment to contain practice sessions and a delayed retention test, which were all conducted on separate days.

To investigate the role of PMd in motor skill learning we coupled brain stimulation with motor-skill acquisition, pairing the delivery of an epoch of excitatory (5 Hz) repetitive TMS (rTMS) with movement task practice. To verify the effects of excitatory rTMS, we also trained a group of individuals who only received inhibitory (1 Hz) rTMS and another cohort who received sham stimulation. Because of the known role of PMd in motor learning and its purported role in the stabilization of newly acquired skills, we hypothesized that excitatory rTMS to PMd would facilitate motor skill consolidation.

## Methods

### Participants

Thirty-two healthy, neurologically intact individuals aged 20 to 38 (14 men and 18 women) enrolled in the experiment (Table 1). All participants gave written informed consent and the protocol was approved by the University research ethics board. Two participants were unable to complete the testing as a result of discomfort during initial motor thresholding with TMS. All participants reported to be right hand dominant; all received left sided rTMS. Participants were not enrolled if 1) they exhibited any frank or clinically evident signs of neurological impairment or disease [25], or 2) they had any color blindness that would impair response ability. Participants were recruited from the University, the local community and the lab database.

**Table 1:** Subject characteristics for the Excitatory, Inhibitory, and Sham groups

	**Excite**	**Inhibit**	**Sham**
**Age (years ± sd)**	24 ± 2	24 ± 1	27 ± 7
**Gender**	6 M, 4 F	3 M, 7 F	5 M, 5 F

### Behavioural task

**Figure 1:**
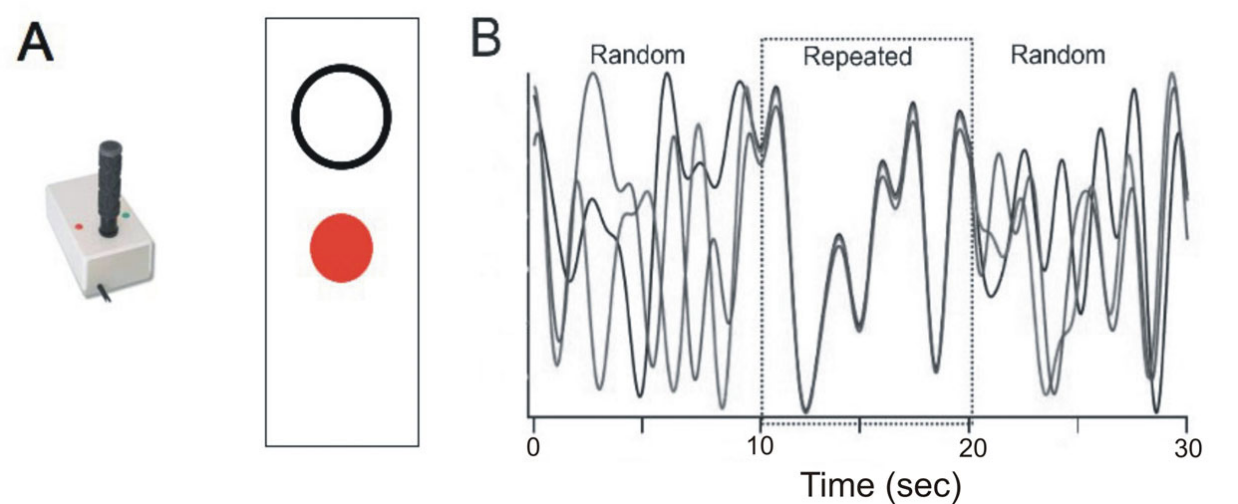
**Illustration of the behavioral task**. A) Continuous tracking of sequences was performed using a joystick; participants were instructed to manipulate the joystick in the vertical direction to track the target (open circle) as accurately as possible. Participant movements appeared as a red, closed circle. B) Continuous tracking trials were constructed from three individual sequences that were joined seamlessly to form one trace. Unknown to participants, the first and last third of each trial (10 s each) were random. The middle third was repeated on every tracking trial. Unlike this illustration, no trace or trail from movement was evident during tacking, only the target and current position of the participant's cursor were visible.

Participants were seated in front of a computer monitor and engaged in continuous tracking of a target moving in a sine-cosine waveform by manipulating a joystick using their right arm [26-29]. The target appeared as a white circle and participant movements were represented as a red dot (Figure 1A). Joystick position sampling and all stimuli were presented at 40 Hz using custom software developed on the LabView platform (v. 7.1; National Instruments Co.).

The pattern of target movement was predefined according to a method modified from Wulf and Schmidt.[26] A unique 33s trial was constructed from one 3s baseline and three 10s sine-cosine segments. One block consisted of ten 33 second trials. Unknown to the participants, the middle third of each tracking pattern was repeated and identical across practice and retention. This pattern was constructed using the polynomial equation as described by Wulf and Schmidt (1997) with the following general form:



The middle (repeated) segment was constructed by using the same coefficients for every trial (Appendix 1). The first and third segments of the tracking pattern were generated randomly using coefficients ranging from 5.0 to -5.0. A different random sequence was used for both the first and third segments for every trial (Figure 1B); however, to ensure uniformity across participants the same set of trials were practiced by all of the participants so that on any given trial the random segments were the same for each participant. In each third of the tracking pattern there were 10 separate reversals in the direction. The trajectories of the target and participants' movements did not leave a trail and thus, participants could not visualize the entire target pattern.

The same trial order was employed for every participant. Participants were not informed of the existence of the repeating sequence but instructed daily to track the target as accurately as possible by controlling the position of the cursor with the joystick.

### TMS

Application of TMS was performed with a 70 mm figure-8 air-cooled coil (Magstim Super Rapid^2^, Magstim Company, Ltd.). The magnetic stimulus had a biphasic waveform with a pulse width of 400 *u*s. During stimulation of both M1 for thresholding and PMd for repetitive stimulation the TMS coil was oriented tangentially to the scalp with the handle pointing back and away from midline at 45 degrees. Prior to the start of the experiment each participant underwent an anatomical MRI scan on a separate day at the UBC 3T MRI Centre (T1 images TE = 5 ms, TR = 24 ms, 40° flip angle, NEX = 1, thickness = 1.2 mm, FOV = 256 mm). These images were imported into Brainsight™ TMS neuronavigation software (Rogue Research Inc.) to allow for stereotaxic registration of the participant's brain with TMS coil for online control of coil positioning.

**Figure 2:**
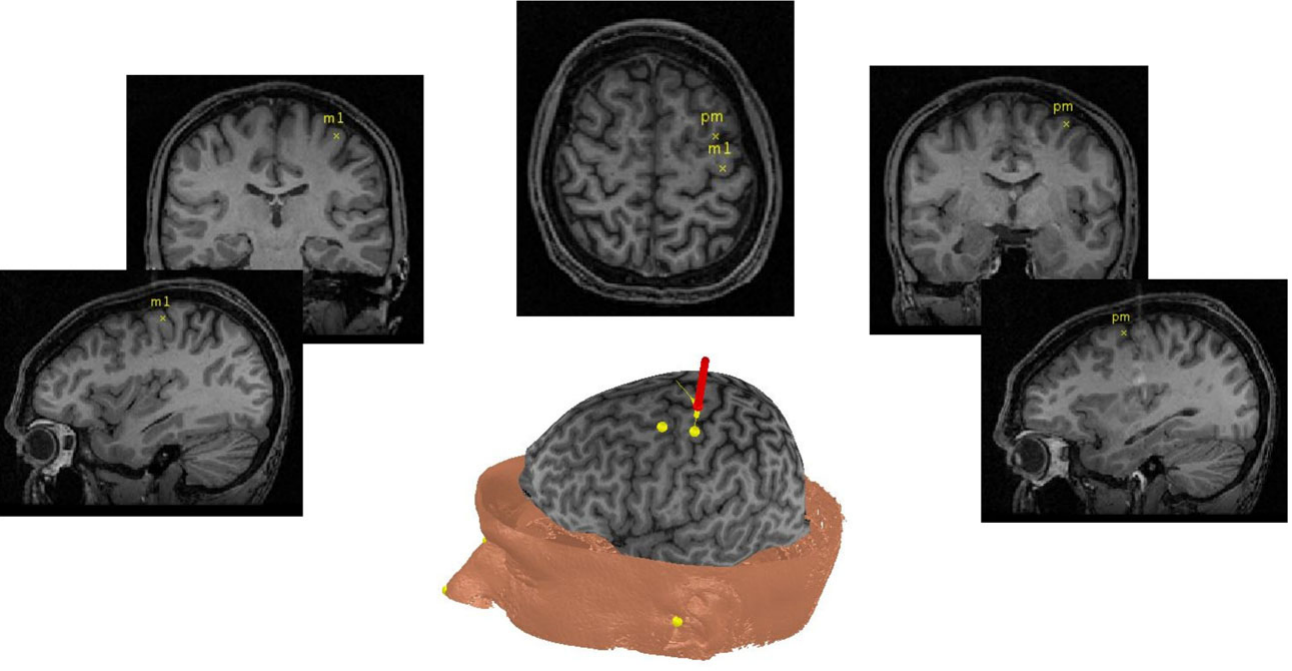
**Illustration of the sterotaxic system and markers that guided TMS coil placement**. Brainsight™ was used to locate primary motor cortex (M1) for resting motor threshold determination and also to subsequently for coil placement over PMd for rTMS. Markers were placed on day 1 of testing to ensure accuracy and repeatability of coil placement and rTMS application across days.

Participants were instructed to remain relaxed throughout the application of rTMS. Surface electromyography (EMG) from participants' right flexor digitorum muscle was monitored through the output screen attached to the transcranial magnetic stimulator (Magstim Super Rapid^2^, Magstim Company, Ltd.). Determination of the location of left primary motor cortex (M1) for resting motor threshold was performed using Brainsight. M1 was identified using the axial scans by locating the "hand knob" and hook MRI images.[30-34] Resting motor threshold (RMT) was determined for each participant, as the percentage of maximal stimulator output to evoke a response of ≥ 50 μV in 5 of 10 trials. The location and trajectory of the coil for this spot was marked using Brainsight™ to minimize variability across subsequent trials and days (Figure 2). Next, the left dorsal pre-motor (PMd) area was marked in Brainsight™ by moving one gyrus forward from the flexor digitorum "hot spot" identified during determination of RMT. The location of PMd was confirmed as the posterior aspect of the middle frontal gyrus (Figure 1).[21,33-38]

Several steps were taken to ensure that stimulation of PMd without M1 during rTMS. First we used a coil that has previously been shown to have a focal enough output to stimulate PMd in isolation. Application of TMS was performed with a 70 mm figure-8 air-cooled coil (Magstim Super Rapid^2^, Magstim Company, Ltd.). Past work has demonstrated that a 70 mm coil can deliver focal stimulation [39] with a current spread small enough, 10 × 10 × 20 mm [31], to stimulate M1 without PMd and vice versa.[40] Second, we oriented our coil over the PMd using anatomic landmarks shown via each individual's T1 MRI to guide us to the posterior aspect of the middle frontal gyrus.[21,35] Once confirmed, the location of PMd and the direction of our stimulation were maintained both within and across sessions by trajectory targeting using BrainSight.

Participants were randomly assigned to one of three groups: 5 Hz excitatory rTMS stimulation (Excite group), 1 Hz inhibitory rTMS stimulation (Inhibit group), or 5 Hz sham stimulation (Sham group). Sham stimulation was performed using a custom sham coil that looks and sounds like an active coil but does not induce any current in the underlying cortex (Magstim Company Ltd.). All participants were naive to TMS measures and were blinded to group assignment. rTMS was performed over the marked spot for left PMd for 15 minutes at 120% of RMT.[21,41] If stimulation at this level caused any visible motor activation intensity was decreased in 5% increments of RMT until there was no longer any motor response. rTMS stimulation intensity over PMd was decreased to eliminate motor response in 16 of 20 participants who received active stimulation: 10 in the Excite group and 6 in the Inhibit group. Across participants rTMS stimulation was never decreased below 100% RMT (Tables 2 &3).

**Table 2:** Mean (standard deviation) RMT from day 1 and day 5 of the experiment by group.

	**Day 1**	**Day 5**	**p-value**
**Excite**	59.3 (7.1)	59.0 (8.3)	.93
**Inhibit**	58.0 (7.6)	59.7 (7.5)	.62
**Sham**	59.5 (9.1)	59.8 (9.0)	.94

Paired t-tests revealed that there were no statistically significant changes in RMT from day 1 to day 5 of the experiment.

**Table 3:** Mean (standard deviation) adjusted %RMT for rTMS from days 1 to 4 of the experiment for the Excite and Inhibit groups.

	**Day 1**	**Day 2**	**Day 3**	**Day 4**
**Excite**	107.0 (7.2)	106.6 (4.8)	104.3 (2.7)	104.9 (2.2)
**Inhibit**	112.1 (8.6)	109.3 (7.8)	107.5 (8.7)	108.0 (8.4)

Because a 15 minute bout of rTMS has been shown to induce approximately a 15-minute after-effect,[42] individuals underwent rTMS first, then immediately practiced the motor task on each of the four practice days. This structure was identical regardless of the rTMS group assignment.

### Design and procedures

The experiment lasted for five days spread over a 2-week timeframe. Days 1–4 were training (rTMS paired with motor task practice). In each of these days participants performed 3 blocks (30 trials) of tracking. Tracking was performed immediately after application of rTMS. Participants were given 2 weeks to complete the entire 5-day experiment, but no more than one day lapsed between day 4 of practice and the retention test for any subject. That is, retention testing always occurred within 48 hours of the last practice session. However, it was necessary to allow days between practice sessions in order to accommodate individual participants' schedules.

To better separate performance effects from more permanent changes in behaviour associated with learning [23], a retention test consisting of 1 block of continuous tracking was given on a separate 5^th ^day. No rTMS was administered at the retention test.

### Repeated sequence awareness

On day 5 following the retention test block, participants were shown 10, 30s blocks (all 3 sequences) of continuous target movement and asked to decide if they recognized any as the pattern that they had seen during practice. Three of the 10 were "true" middle sequence i.e., the same as the repeated practice pattern; 7 were foils. Individuals who identified the repeated sequence at a better than chance rate, i.e., 2 of 3 repeated sequences identified correctly as being recognized and 4 of 7 novel, random epochs identified correctly as never having been seen before, were considered to have gained explicit awareness of the repeating sequence.

### Outcome measures

Motor performance was evaluated across practice and retention. Our analysis considered changes in root mean squared error (RMSE; Appendix 2), which reflects overall tracking error in the kinematic pattern and is the average difference between the target pattern and participant movements. This score was calculated separately for random and repeating sequences on every tracking trial and averaged by block (every 10 trials)[27,28,43]. Comparison between RMSE from the repeated and random sequences reflects sequence specific learning. This measure was used to evaluate reductions in tracking errors across practice and at retention.

To investigate the possibility that rTMS stimulated off-line motor learning we calculated a change score to reflect the difference in tracking error at the end of practice with that at the retention test. This computation was performed for both repeated and random sequences. We assume that continued further decrease in tracking error (RMSE) between the last practice block and the retention test reflects off-line motor learning associated with consolidation [6,7].

### Statistical analyses

Prior to running analyses of variance on our motor practice and learning data the normality of distribution was assessed with a Kolmogorov-Smirnov test. The data were normally distributed. Overall, our analyses were conducted in three steps. First, we considered performance related changes across the four experimental days when practice was paired with rTMS. Second, we assessed motor learning at the retention test on day 5. Third, we assessed offline learning. We defined off-line learning related gains according to Robertson (2004, 2006) as the difference in RMSE in between the last block of practice on day 4 (when rTMS was last delivered) and the retention test on day 5 when there was no rTMS. Our retention test was delivered within 48 hours of the last practice day to ensure that we assessed off-line learning within the accepted time frame.[7,44]

Acquisition practice. Performance of the repeated sequence during practice was examined using two factor (Group [Excite, Inhibit, Sham] X Block [1-12]) repeated measures ANOVA. This analysis was performed separately with repeated sequence RMSE and random sequence RMSE as the dependent variables.

Retention. Motor learning at retention was examined via a Group [Excite, Inhibit, Sham] by Sequence [Random, Repeated] repeated measures ANOVA with RMSE or tracking error as the dependent measure. A Bonferroni correction was used for post-hoc tests to determine the locus of significant group by sequence interactions. Off-line motor learning was assessed via a one-way ANOVA using the change score from the last block of practice to the retention test as the dependent measure. This test was performed separately for random and repeated sequence change scores.

## Results

Overall, our data demonstrate three main results. First, regardless of stimulation condition tracking error as reflected by RMSE decreased with practice. Second, at the retention test all groups showed motor learning of the repeated sequence; however, the largest amount of change between repeated and random sequence tracking error was shown by the Excite group. Third, consideration of gains made in tracking accuracy between the last block of practice and the retention test demonstrate off-line motor learning for the Excite group but not for the Inhibit or Sham groups.

### Acquisition practice

**Figure 3:**
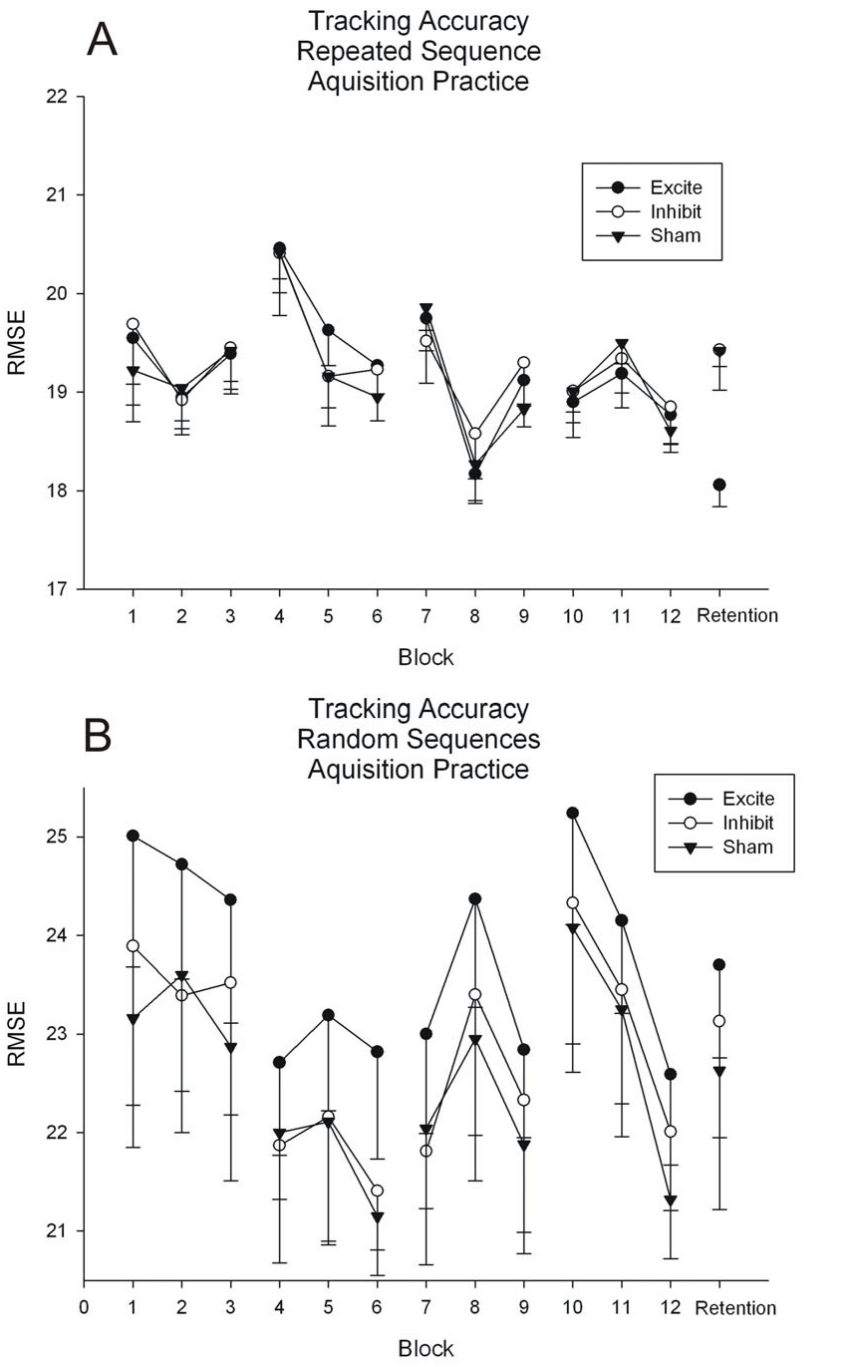
**Twelve blocks of sequence tracking were performed across four days of practice (3 blocks per day; each block consisted of 10 trials of the 30 track)**. Root mean square error (RMSE) for repeated and random sequence tracking was calculated. A) RMSE for repeated sequences across practice and at the retention test. B) RMSE for random sequences across practice and at the retention test. Data are mean RMSE ± standard error of the mean (SEM).

All groups improved performance on the repeating sequence across practice as demonstrated by a main effect of Block for repeating sequence tracking error (F(11, 286) = 15.23, p = .000; Figure 3A). In addition, non-specific improvements in tracking that reflect improved motor control during random sequence tracking also was demonstrated by a main effect of Block (F(11,286) = 11.31, p = .000; Figure 3B). There were no significant interactions for either random or repeated sequence tracking data over practice.

### Retention

**Figure 4:**
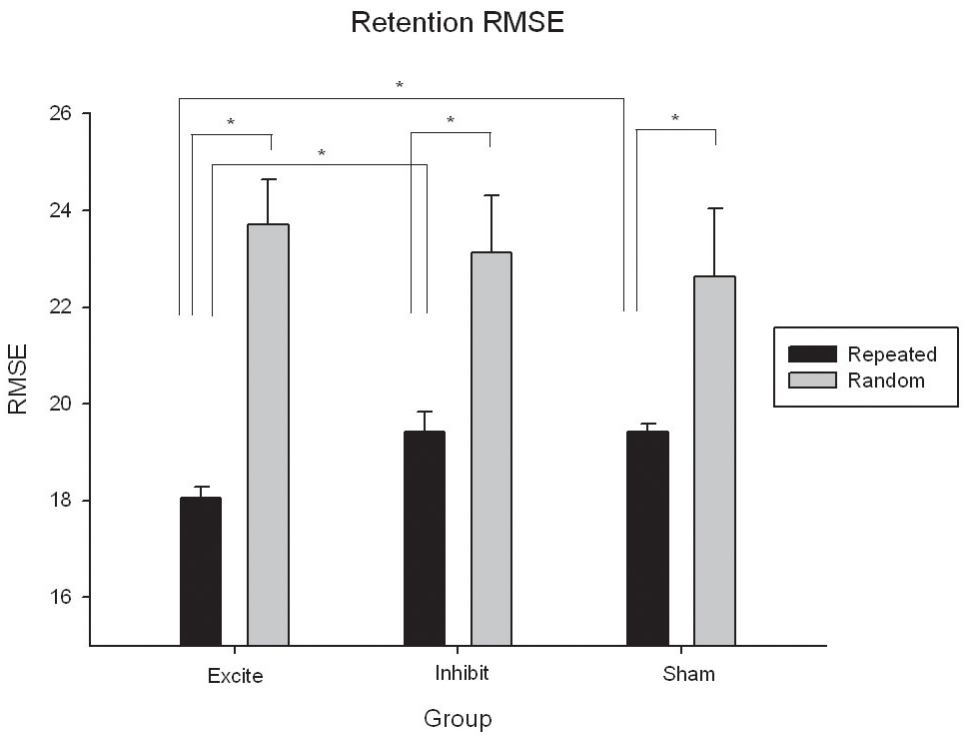
**RMSE for repeated and random sequences at the retention test**. All groups showed sequence specific motor learning as demonstrated by significantly lower RMSE for repeated as compared to random sequences at retention. However, individuals in the Excite group showed even lower tracking error for repeated sequences that those in the Inhibit or Sham stimulation groups. Data are mean RMSE ± SEM.

All groups demonstrated motor learning at retention as shown by a main effect of Sequence that illustrated a significant difference between tracking error for repeated and random sequences (F(1,26) = 99.28, p = .000). More interesting, was a Group by Sequence interaction (F(2,26) = 4.257, p = .003). Post-hoc testing revealed that the Excite group made less tracking error than then Inhibit group (p = .002) or Sham group (p = .012) during repeated sequence tracking but not during random sequence tracking at retention (Figure 4).

### Off-line learning

**Figure 5:**
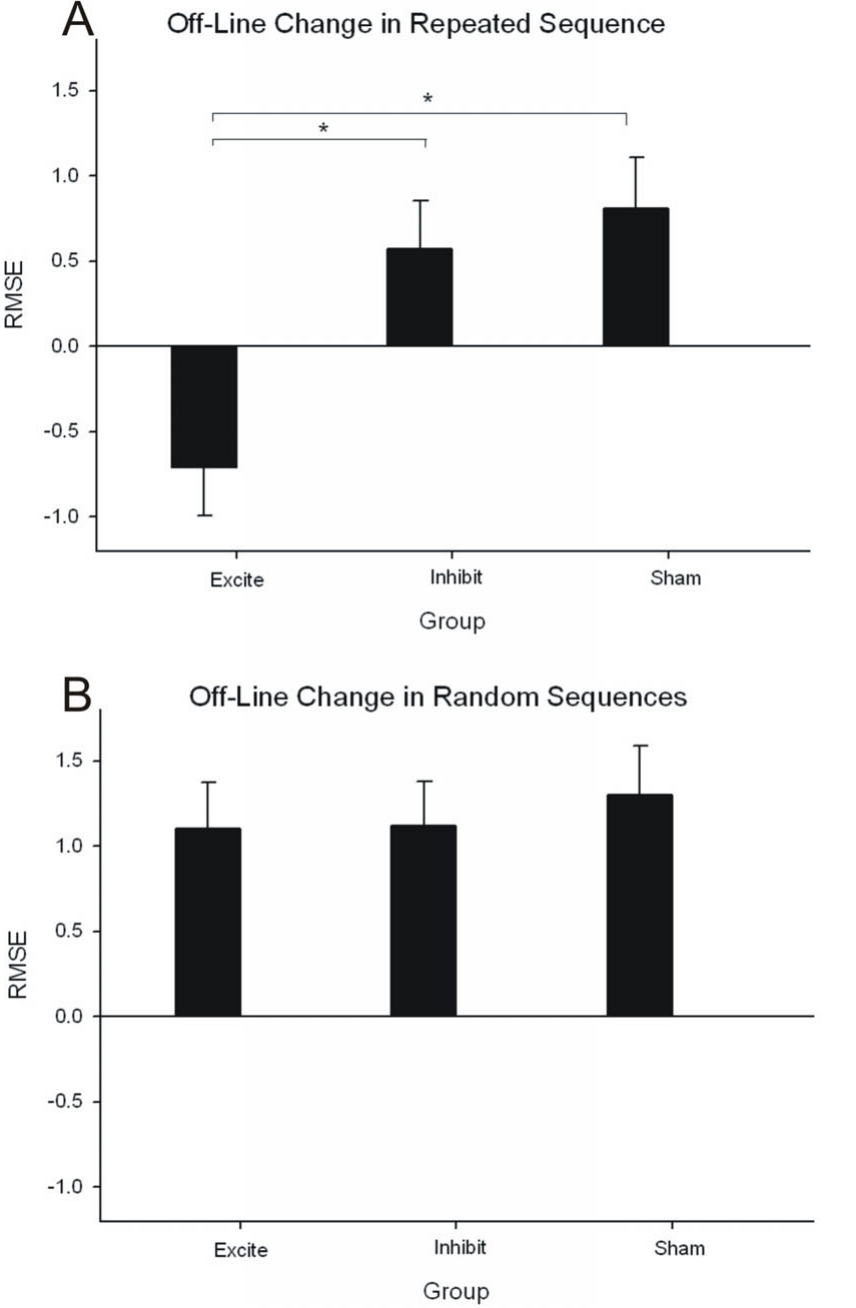
**Change in tracking error (RMSE) between the last block of sequence practice and the retention test by group for repeated and random sequences**. A) Sequence specific off-line learning was evident for the Excite group as demonstrated by the continued *decrease *in tracking error from practice to retention (show by negative numbers). This was not the case for the Inhibit or Sham stimulation groups who showed slight losses in tracking accuracy as evidenced by positive changes from practice to retention. B) No non-specific generalized motor control improvements occurred off-line. For random sequences slightly higher tracking error was shown at retention as compared to practice for all three groups. Data are mean change in RMSE ± standard error of the mean (SEM).

Between group differences in consolidation that occurred off-line were illustrated by a significant one-way ANOVA using the repeated sequence change scores from the groups as the dependent measure (F(91,26) = 8.32, p = .002). Consolidation of motor learning occurred off-line for the Excite group as demonstrated by the continued decrease in tracking error that occurred between the end of practice and the retention test. This finding is contrasted to the Inhibit and Sham groups who both showed slightly worse tracking error at retention as compared to the end of practice (Figure 5A). There were no between group differences in change scores for random sequence tracking (Figure 5B).

### Explicit knowledge

None of the experimental groups gained explicit knowledge of the repeating sequence as demonstrated by the ability to identify the repeating sequence during recognition tests on the final day at chance.

## Discussion

Even single sessions of motor practice can lead to long-term storage of movement representations in the brain [5]. It is now clear that after practice has ended the functional properties and representation of skilled movement continues to evolve in the brain [4-7]. These changes are evident in the gradual development of resistance to interference from other behaviours as time passes after task practice [4,5]. In some cases motor skills are not merely stabilized but can be improved through this consolidation process [6,7]. Indeed, this is what we discovered when we paired 5 Hz excitatory rTMS to left PMd with motor task practice; motor skill continued to improve off-line after practice. Conversely, participants who practiced the motor task and received either inhibitory or sham stimulation showed only memory stabilization, there was no further between session improvement, but rather a relative preservation in motor skill level acquired via practice [7]. Though these two forms of memory consolidation are not mutually exclusive, our data suggest that PMd has a role in off-line motor skill enhancement. Importantly, we separated the short-term effects of practice from more permanent changes in behaviour demonstrated at retention by performing these tests on different days [23]. This experimental feature allowed us to view off-line learning in the Excite group without any interference effects from practice [7].

Critically, the off-line motor consolidation demonstrated by the Excite group was related to sequence-specific motor learning rather than to generalized improvements in motor control associated with task practice. Illustration of this point is evident in the difference in tracking error across groups for the repeated sequence at retention (Figure 4); the three groups demonstrated equivalent performance on random sequences at the same time. Random sequence tracking reflects generalized motor execution whereas repeated sequence performance shows motor learning [27,28,43]. Thus, the role of PMd in motor consolidation in the present work related to implicit sequence-specific learning rather than an overall improvement in the generalized ability to track continuous sequences.

There has been debate as to whether PMd activity relates to motor learning [22] or to the recall of already learned movements [5,20]. Our data support the hypothesis that PMd activity facilitates motor learning, specifically by aiding memory consolidation. Two features of our data support our conclusion. Critically, excitatory stimulation to PMd promoted off-line learning. We expected that if PMd played a role in recall of learned movements rather than in motor learning, we would have noted memory stabilization rather than off-line improvements. Second, the improvements associated with excitatory stimulation to PMd were sequence-specific and not simply related to generalized motor control improvements.

The influence of PMd activity on motor learning and memory consolidation likely operated though a network of brain regions. PMd is ideally situated to impact a broad range of cortico-cortical and cortic-subcortical networks. On-line rTMS-fMRI imaging has shown that excitatory stimulation of PMd increases the BOLD signal both locally (in PMd, PMv, supplementary motor area, somatosensory cortex, and cingulate motor area) and distantly (in contralateral PMd, cerebellum, putamen and caudate; [45]). Further, these rTMS driven modifications in hemodynamics occur even in the absence of overt motor responses. This pattern of brain activation associated with rTMS to PMd reflects the known anatomical and functional connectivity amongst these regions [14,15,45]. Though we cannot ascribe the offline learning we documented to any single region within this broad network, it is evident that 5 Hz rTMS stimulated motor memory consolidation most likely via up-regulating at least some elements of both local and distantly connected brain regions.

We expected that the Inhibit group might have demonstrated worse behaviour than those participants in the Sham stimulation condition. However, it may be that the positive effects of practice on accuracy of motor tracking performance countered the impact of 1 Hz stimulation. Indeed, we and others [26-28,46] have shown that motor task practice of continuous tracking tasks may be well learned over as few as three practice sessions. It is possible that in the present study the effects of 1 Hz TMS was either overcome by motor practice or that the network of brain regions associated with motor learning [5,21,45] was able to compensate for less PMd function following inhibitory stimulation.

Though sleep may have played a role in the consolidation we noted across our experimental groups it cannot explain the lower tracking error for repeated sequences shown only by the Excite group at retention. Each of our groups slept in between the last practice day and the retention test; conferring the benefit of sleep on motor skill consolidation regardless of group assignment. Further, past work has demonstrated that off-line improvements in implicit motor learning in young, healthy controls are not sleep dependent [47]. Instead, sleep related improvements in motor skill may develop equally well over the day as they do over the night [44]. Thus, we do not believe that the sleep-induced benefits that are associated with consolidation can account for our findings.

It is also unlikely that differences in explicit knowledge explain any of our group differences across practice or at retention; none of the groups gained explicit awareness of the repeating sequence. In addition, past work [27,48] has not shown a benefit of explicit knowledge for motor learning of tracking tasks. Based on the results of our explicit tests we are confident that our data reflect changes associated with the implicit motor learning system.

We were surprised at the large number (n = 16) of individuals who required TMS intensity to be reduced owing to inadvertent motor twitching during PMd stimulation. These individuals were from the Excite and Inhibit groups alike. One possible explanation is that the threshold for stimulating primary motor cortex (from which we derived our resting motor threshold) is not the same as the threshold in other brain regions [45,49]. Specifically, it is possible that PMd has a lower threshold for stimulation than M1; thus, stimulation of PMd may have either activated M1 via PMd-M1 connections or recruited descending tracts from PMd that normally would not fire at lower intensities. Future work will have to endeavor to develop methods for thresholding stimulation intensity more appropriately for regions outside motor cortex.

## Conclusion

Taken together, our data support a role for PMd in motor memory consolidation through the process of off-line learning. In addition, our findings support the concept that motor memory consolidation may take two distinct forms (off-line improvement and memory stabilization) and that these processes may be dissociated during learning of the same task [6,7]. Though it is likely that 5 HZ rTMS increased activation in a network of brain regions, we did note a strong influence on excitatory stimulation on memory consolidation suggesting a role for PMd in motor learning. Importantly, the positive effect of 5 HZ rTMS to PMd was directly related to sequence-specific motor learning and had little effect on generalized motor control during continuous tracking.

## Authors' contributions

LB conceived of the study, designed the experiment, and programmed the task and analyses. ML coordinated and collected the TMS and behavioral data. Both LB and ML drafted the manuscript, read and approved the final manuscript.

## Appendix 1

b_0 _= 2.0, a_1 _= -4.0, b_1 _= 3.0, a_2 _= -4.9, b_2 _= -3.6, a_3 _= 3.9, b_3 _= 4.5, a_4 _= 0.0, b_4 _= 1.0, a_5 _= -3.8, b_5 _= -0.5, a_6 _= 1.0, and b_6 _= 2.5

## Appendix 2



x_*i *_= participant's position in degrees at time 1, T_*i *_= target position at time 1, n = the number of samples for the participant's trajectory array
